# Knowledge levels of doctors and nurses working in surgical clinics about nutrients and food supplements, a multicentre descriptive study

**DOI:** 10.1186/s12912-024-01968-z

**Published:** 2024-04-25

**Authors:** Aslı Emine Büyükkasap, Gülay Yazıcı

**Affiliations:** 1 Gazi University Health Research and Application Center, Ankara, Turkey; 2 Faculty of Health Sciences, Ankara Yıldırım Beyazıt University, Ankara, Turkey

**Keywords:** Nutrients and food supplements, Level of knowledge, Surgery, Doctor, Nurse

## Abstract

**Purpose:**

The use of nutrients and food supplements is increasing worldwide. Nutrients and food supplements frequently used in the surgical period may cause complications and side effects. This study was conducted to determine the level of knowledge about sixty-one nutrients and food supplements among doctors and nurses working in surgical clinics.

**Design:**

A multicentre descriptive, quantitative, cross-sectional study.

**Methods:**

The study was conducted between 15 February and 31 May 2022 with a total of 410 participants, including 143 doctors and 267 nurses, working in the surgical clinics of 8 hospitals, including public, university and private hospitals, within the borders of one province in Turkey. Data were collected face-to-face using a questionnaire developed by the researchers, which included descriptive characteristics of the doctors and nurses and questions about sixty-one nutrients and food supplements.

**Results:**

The median overall success score of the doctors and nurses participating in the study regarding the use of nutrients and food supplements was 3.20 out of 100 points, the median success score of complications and side effects of nutrients and food supplements in the surgical period was 7.06 out of 33 points, the median success score for discontinuation of nutrients and food supplements prior to surgery was 0.21 out of 16 points, and the median success score for drug interactions of nutrients and food supplements was 1.70 out of 51 points. In addition, it was found that the overall success scores of doctors and nurses regarding nutrients and food supplements increased statistically significantly with increasing age and working years. The total success score of doctors and nurses who received training in nutrients and food supplements was statistically significantly higher than that of doctors and nurses who did not receive training.

**Conclusion:**

In conclusion, it was found that the level of knowledge of nutrients and food supplements among doctors and nurses working in surgical clinics was less than half or even close to zero. Therefore, it is recommended that training on nutrients and food supplements should be included in the undergraduate and postgraduate education of doctors and nurses in order to prevent complications, side effects, drug interactions and patient safety related to the use of nutrients and food supplements in the surgical period.

**Supplementary Information:**

The online version contains supplementary material available at 10.1186/s12912-024-01968-z.

## Introduction

Plants, medicinal herbs, foods, and their derivatives have been used for centuries to prevent, treat, and maintain physical and mental health [1, 2]. With the advancement of the modern pharmaceutical industry, these natural remedies have been transformed into nutrients and food supplements through physical and biological processes [3]. These products comprise of a variety of vitamins, minerals, amino acids, probiotics, or herbal components [4, 5]. Nutrients and food supplements that are used to support daily nutrition are extensively used globally [2, 6, 7]. As per the World Health Organisation, around 80% of individuals in developing countries use nutrients and food supplements [8]. In developed countries, the use of nutrients and food supplements varies widely, with Spain at 41%, Canada at 70%, Australia at 82%, the United States of America at 35%, and Turkey at 53% [9–11].

The promotion of these products in mass media [12–15] and recommendations from friends and family [1, 12, 14–17], combined with their availability without prescription and affordability [16, 18, 19], contribute to their increased use [14, 16, 20]. Nutrients and food supplements are often preferred for improving health and well-being, alleviating symptoms associated with chronic diseases [10], cancer treatment [10, 21], pregnancy [22], and the surgical period [23–25].

Nevertheless, their use can cause drug interactions and serious complications during surgery [24, 26, 27]. Nutrients and food supplements alter the efficacy of anticoagulants and antiplatelets [28], increase the efficacy of antihypertensive and antidiabetic drugs [29], interact with corticosteroids, central nervous system depressants, opioid analgesics [30] and anaesthetics [31, 32] and increase the efficacy of sedatives and tranquillisers [13]. These supplements may also cause prolonged sedation [31, 33, 34], delayed recovery from anaesthesia [15, 29], bleeding [15, 31, 33–35], coagulation disorders [34, 35], cardiac problems [29, 34], fluid-electrolyte imbalances [34, 36], hypoglycaemia [15, 34], affecting the need for analgesics after surgery [31], transplant rejection, irreversible side effects such as kidney [29] and liver toxicity [29, 37] and even death. To prevent complications, side effects, and drug interactions, it is recommended to stop taking nutrients and food supplements two weeks prior to surgery [31, 32, 35, 38–45]. Awareness and knowledge level of doctors and nurses are very important in order to prevent, recognise and treat complications related to nutrients and food supplements [25–27, 46]. Upon examination of the literature, it was found that there is a lack of studies on doctors' knowledge of nutrients and food supplements, and the existing studies indicate that their knowledge on the subject is inadequate [47, 48]. Similarly, no studies were found regarding the knowledge level of nurses on nutrients and food supplements.

Doctors and nurses working in surgical clinics should have the necessary awareness and knowledge to prevent, recognise, and treat complications related to nutrients and food supplements [25–27, 46]. This study aimed to determine the level of knowledge of doctors and nurses working in surgical clinics regarding complications and side effects of sixty-one nutrients and food supplements, withdrawal periods, and drug interactions.

## Methods

### Type of research

The study was conducted in a descriptive, quantitative, cross-sectional manner to determine the level of knowledge about sixty-one nutrients and food supplements among doctors and nurses working in surgical clinics.

### Research population and sample

The population of the research consists of a total of 1,537 people, including 700 doctors and 837 nurses, working in the surgical clinics of 8 hospitals (6 public, 1 university and 1 private hospital) within the borders of a province in Turkey that approved the study. The sample calculation of the study was made with 50% unknown frequency and 95% confidence interval with type 1 error 0.05, and it was calculated that at least 307 participants should be reached. When the sample size was stratified separately for doctors and nurses, it was found that 140 doctors and 167 nurses needed to be reached. Criteria for inclusion in the study; working as a staff doctor or nurse in surgical clinics, volunteering to participate in the research, working in adult surgical clinics. Exclusion criteria from the study were as follows; not volunteering to participate in the research, not answering the entire questionnaire, working in surgical intensive care units, working in a pediatric surgery clinic, not being a permanent employee of the clinic where the research is conducted, being in the clinic due to rotation, working in the clinic on a temporary basis due to day or night shifts. The study was completed with a total of 410 people, including 143 doctors (6 from private hospitals, 30 from university hospitals, 107 from public hospitals) and 267 nurses (25 from private hospitals, 45 from university hospitals, 197 from public hospitals), who agreed to participate in the study, gave written consent, and completed the entire questionnaire. Doctors and nurses working in hospitals do not receive training in nutrients and food supplements.

### Data collection tools

The research used a questionnaire-interview method to collect data. This questionnaire-interview was designed for this study by the researchers utilizing the literature (Supplementary Material). The questionnaire used for data collection consists of four parts. The first part includes the descriptive characteristics of the doctors and nurses, the second part includes the complications and side effects seen during the surgical period due to the use of nutrients and food supplements, the third part includes the duration of discontinuation of nutrients and food supplements before the surgical period, and the fourth part includes questions about drug interactions of nutrients and food supplements.

#### Section 1: descriptive characteristics of doctors and nurses

This section was developed by the researcher based on the literature [1, 10, 11, 14, 16, 20, 49, 50]. This section consists of a total of 14 questions about doctors and nurses, including age, sex, education level, marital status, occupation, total years of professional experience, clinic where they work, total years of experience in the department where they work, status of training in nutrients and food supplements, status of questioning patients about the use of nutrients and dietary supplements, status of postponement of surgery due to the use of nutrients and dietary supplements, and number of patients for whom surgery was postponed.

#### Section 2: questions related to complications and side effects during the surgical period due to the use of nutrients and food supplements

This section was developed by the researcher based on the literature [4, 13, 24, 28, 29, 32, 35, 42, 44, 50–74].

This section consists of questions about complications and side effects caused by 61 foods and nutrients and food supplements (garlic, onion, lemon, nettle, parsley, red pepper (capsicum), chia seed, celery, rosemary, sage, thyme, St. John's wort, turmeric, ginger, hawthorn, aloe vera, black tea, green tea, linden, lavender, chamomile, echinacea, calendula, clove, dandelion, ginseng, ginkgo biloba, cherry, blueberry, horse chestnut, liquorice, valerian, red clover, anise seed, ephedra, kava, fenugreek, black cohosh, burdock, cat's claw, mistletoe, hops, passionflower, bitter melon, devil's claw, coenzyme Q10, vitamin E, vitamin C, vitamin B12, vitamin D, fish oil, calcium, magnesium, iron, zinc, folic acid, alpha-lipoic acid, L-arginine, sports supplements, probiotics, weight-loss products).

These questions consist of a total of four questions: bleeding, fluid-electrolyte imbalance, hepatotoxic effects and effects on blood glucose levels. Each box in which the answers to the questions are scored corresponds to a response option. The answer options for the questions assessing bleeding are "increases", "decreases", "has no effect" and "don't know"; the answer options for the question assessing fluid-electrolyte imbalance are "does", "has no effect" and "don't know", the answer choices for the hepatotoxic effect question are 'does', 'has no effect' and 'don't know', and the answer choices for the blood glucose effect question are 'increases', 'decreases', 'has no effect' and 'don't know'.

For example, question 1 consists of four questions about the effects of garlic on bleeding, fluid-electrolyte imbalance, hepatotoxic effects, and blood glucose levels. When the literature was reviewed, it was found that garlic affects bleeding [44] and blood sugar [50, 53]. However, no information was found in the literature on fluid-electrolyte imbalance and hepatotoxic effects. For this reason, the questions related to fluid-electrolyte imbalance and hepatotoxic effects were excluded from the evaluation and the effects on bleeding and blood glucose levels were evaluated. In this context, questions on 61 nutrients and food supplements that could not be found in the literature were excluded. Table 1 below shows the nutrients and food supplements that were excluded in this section. As a result, a total of 244 questions were asked in this section and 131 questions were evaluated in line with the literature (Table 1).

**Table 1:** Questions excluded from the evaluation in the section related to complications and side effects seen in the surgical period due to the use of nutrients and food supplements.

**Number of Questions Excluded from the Evaluation (Total 113 Questions)**	**Questions on Nutrients and Food Supplements Excluded from Evaluation**
Bleeding (9 questions)	8.Celery, 19. Linden, 43. Passionflower, 54. Iron, 56. Folic acid, 57. Alpha-lipoic acid, 58. L-arginine, 59. Sports supplement, 60. Probiotics
Fluid-Electrolyte Imbalance (29 Questions)	1. Garlic, 6. Red Pepper (Capsicum), 7. Chia seeds, 8. Celery, 9. Rosemary, 13. Turmeric, 15. Hawthorn, 16. Aloe vera, 20. Lavender, 21. Chamomile, 22. Echinacea, 25. Dandelion, 29. Blueberry, 30. Horse chestnut, 33. Red clover, 34. Anise seed, 38. Black cohosh, 39. Burdock, 40. Cat's claw, 41. Mistletoe, 42. Hops, 44. Bitter melon, 45. Devil's claw, 46. Coenzyme Q10, 47. Vitamin E, 49. Vitamin B12, 51. Fish oil, 56. Folic acid, 57. Alpha-lipoic acid
Hepatotoxic Effect (45 questions)	1. Garlic, 2. Onion, 3. Lemon, 4. Nettle, 5. Parsley, 6. Red pepper (Capsicum), 7. Chia seeds, 8. Celery, 9. Rosemary, 12. St. John's wort, 13. Turmeric, 24. Ginger, 15. Hawthorn, 16. Aloe vera, 17. Black tea, 19. Linden, 20. Lavender, 21. Chamomile, 23. Calendula, 24. Clove, 25. Dandelion, 26. Ginseng, 27. Ginkgo biloba, 28. Cherry, 29. Blueberry, 30. Horse chestnut, 33. Red clover, 34. Anise seed, 35. Ephedra, 37. Fenugreek, 39. Burdock, 40. Cat's claw, 42. Hops, 43. Passionflower, 44. Bitter melon, 45. Devil's claw, 46. Coenzyme Q10, 47. Vitamin E, 48. Vitamin C, 49. Vitamin B12, 50. Vitamin D, 51. Fish oil, 57. Alpha-lipoic acid, 58. L-arginine, 60. Probiotics
Blood Glucose Level (30 questions)	5. Parsley, 6. Red Pepper (Capsicum), 7. Chia Seeds, 11. Thyme, 12. St. John's Wort, 13. Turmeric, 15. Hawthorn, 19. Linden, 20. Lavender, 21. Chamomile, 22. Echinacea, 23. Calendula, 24. Clove, 32. Valerian, 34. Anise Seed, 38. Black cohosh, 40. Cat's claw, 42. Hops, 43. Passionflower, 46. Coenzyme Q10, 47. Vitamin E, 48. Vitamin C, 49. Vitamin B12, 51. Fish oil, 53. Magnesium, 54. Iron, 55. Zinc, 56. Folic acid, 58. L-arginine, 60. Probiotics

#### Section 3: questions related to the duration of discontinuation of nutrients and food supplements before the surgical period

This section was developed by the researcher based on the literature [31, 32, 35, 38–42, 44, 45, 50, 52, 75]. This section consists of a total of 61 questions about when to discontinue nutrients and food supplements (garlic, onion, lemon, nettle, parsley, red pepper (capsicum), chia seed, celery, rosemary, sage, thyme, St. John's wort, turmeric, ginger, hawthorn, aloe vera, black tea, green tea, linden, lavender, chamomile, echinacea, calendula, clove, dandelion, ginseng, ginkgo biloba, cherry, blueberry, horse chestnut, liquorice, valerian, red clover, anise seed, ephedra, kava, fenugreek, black cohosh, burdock, cat's claw, mistletoe, hops, passionflower, bitter melon, devil's claw, coenzyme Q10, vitamin E, vitamin C, vitamin B12, vitamin D, fish oil, calcium, magnesium, iron, zinc, folic acid, alpha-lipoic acid, L-arginine, sports supplements, probiotics, weight-loss products) in the preoperative period. Answers to these questions are sought as ".... should be discontinued weeks ago", ".... should be discontinued days ago (the period less than seven days will be written)", ".... should be discontinued hours ago (the period less than twenty-four hours will be written)", "I do not know the answer". The answer of the participants who answered "there is no need to cut" was noted as "0 hours".

#### Section 4: Drug interaction questionnaire for nutrients and food supplements

This section was developed by the researcher based on the literature [13, 26, 29, 31, 42–44, 50, 51, 53, 56, 76–84]. It consists of questions on the interaction of nutrients and food supplements (garlic, onion, lemon, nettle, parsley, red pepper (capsicum), chia seed, celery, rosemary, sage, thyme, St. John's wort, turmeric, ginger, hawthorn, aloe vera, black tea, green tea, linden, lavender, chamomile, echinacea, calendula, clove, dandelion, ginseng, ginkgo biloba, cherry, blueberry, horse chestnut, liquorice, valerian, red clover, anise seed, ephedra, kava, fenugreek, black cohosh, burdock, cat's claw, mistletoe, hops, passionflower, bitter melon, devil's claw, coenzyme Q10, vitamin E, vitamin C, vitamin B12, vitamin D, fish oil, calcium, magnesium, iron, zinc, folic acid, alpha-lipoic acid, L-arginine, sports supplements, probiotics, weight-loss products) with the drug groups antihypertensive, anticoagulant, anaesthetic, analgesic, corticosteroid, antidiabetic, antidepressant. Each box corresponds to a response option and the options are 'increases', 'decreases', 'has no effect' and 'don't know'.

For example, there are 7 questions assessing the interaction of garlic with the following drug classes: antihypertensive, anticoagulant, anaesthetic, analgesic, corticosteroid, antidiabetic, antidepressant. There is information in the literature that garlic interacts with antihypertensive [44], anticoagulant [13], anaesthetic [31], analgesic [13, 76], antidiabetic and antidepressant [50] drug groups. However, this question was excluded from the analysis as there was no information on the interaction with the corticosteroid group. Therefore, questions about 6 groups of drugs with which garlic interacts were analysed. In this context, questions about 61 nutrients and food supplements that could not be found in the literature were excluded. Table 2 shows the nutrients and food supplements that were excluded from the assessment in this section. As a result, a total of 427 questions were asked in this section and 201 questions were evaluated in accordance with the literature (Table 2).

**Table 2:** Questions excluded from the evaluation in the section on drug interactions of nutrients and food supplements

**Number of Excluded Questions (Total 226 questions)**	**Questions on Nutrients and Food Supplements Excluded from Evaluation**
**Anti-Hypertensives (24 questions)**	13. Turmeric, 16. Aloe vera, 19. Linden, 20. Lavender, 21. Chamomile, 22. Echinacea, 24. Clove, 25. Dandelion, 28. Cherry, 32. Valerian, 33. Red clover, 42. Hops, 43. Passionflower, 44. Bitter melon, 47. Vitamin E, 48. Vitamin C, 49. Vitamin B12, 50. Vitamin D, 51. Fish oil, 55. Zinc, 56. Folic acid, 57. Alpha-lipoic acid, 60. Probiotics, 61. Weight-loss products
**Anticoagulants (9 questions)**	19. Linden, 32. Valerian, 43. Passionflower, 54. Iron, 56. Folic acid, 57. Alpha-lipoic acid, 58. L-arginine, 59. Sports supplement, 60. Probiotics
**Anaesthetics (37 questions)**	2. Onion, 4. Nettle, 6. Red pepper (capsicum), 7. Chia seeds, 9. Rosemary, 11. Thyme, 16. Aloe vera, 17. Black tea, 19. Linden, 24. Clove, 25. Dandelion, 26. Ginseng, 28. Cherry, 29. Blueberry, 30. Horse chestnut, 31. Licorice root, 33. Red clover, 34. Anise seed, 37. Fenugreek, 38. Black cohosh, 39. Burdock, 40. Cat's claw, 41. Mistletoe, 44. Bitter melon, 45. Devil's claw, 46. Coenzyme Q10, 47. Vitamin E, 48. Vitamin C, 49. Vitamin B12, 50. Vitamin D, 51. Fish oil, 52. Calcium, 53. Magnesium, 54. Iron, 56. Folic acid, 57. Alpha-lipoic acid, 58 .L-arginine
**Analgesics (42 questions)**	2. Onion, 3. Lemon, 5. Parsley, 7. Chia seeds, 9. Rosemary, 10. Sage, 11. Thyme, 14. Ginger, 15. Hawthorn, 17. Black tea, 19. Linden, 20. Lavender, 22. Echinacea, 23. Calendula, 24. Clove, 25. Dandelion, 26. Ginseng, 28. Cherry, 29. Blueberry, 30. Horse Chestnut, 33. Red Clover, 34. Anise Seed, 35. Ephedra, 38. Black cohosh, 39. Burdock, 40. Cat's claw, 41. Mistletoe, 42. Hops, 44. Bitter melon, 46. Coenzyme Q10, 47. Vitamin E, 48. Vitamin C, 49. Vitamin B12, 50. Vitamin D, 51. Fish oil, 52. Calcium, 53. Magnesium, 55. Zinc, 57. Alpha-lipoic acid, 58. L-arginine, 59. Sports supplement, 60. Probiotics
**Corticosteroids (57 Questions)**	1. Garlic, 2. Onion, 3. Lemon, 4. Nettle, 5. Parsley, 6. Red Pepper (Capsicum), 7. Chia seeds, 8. Celery, 9. Rosemary, 10. Sage, 11. Thyme, 12. St. John's Wort, 13. Turmeric, 14. Ginger, 15. Black tea, 18. Green tea, 19. Linden, 20. Lavender, 21. Chamomile, 22. Echinacea, 23. Calendula, 24. Clove, 25. Dandelion, 26. Ginseng, 27. Gingko Biloba, 28. Cherry, 29. Blueberry, 30. Horse Chestnut, 32. Valerian, 33. Red Clove, 34. Anise Seed, 35. Ephedra, 36. Kava, 37. Fenugreek, 38. Black Cohosh, 39. Burdock, 40. Cat's Claw, 41. Mistletoe, 42. Hops, 43. Passionflower, 44. Bitter Melon, 45. Devil's Claw, 46. Coenzyme Q10, 47. Vitamin E, 48. Vitamin C, 49. Vitamin B12, 50. Vitamin D, 51. Fish Oil, 52. Calcium, 53. Magnesium, 54. Iron, 55. Zinc, 56. Folic Acid, 57. Alpha-Lipoic Acid, 58. L-Arginine, 59. Sports Supplement, 60. Probiotics
**Anti-diabetics (23 questions)**	15. Hawthorn, 19. Linden, 20. Lavender, 21. Chamomile, 22. Echinacea, 23. Calendula, 24. Clove, 28. Cherry, 32. Valerian, 34. Anise Seed, 36. Kava, 40. Cat's Claw, 42. Hops, 46. Coenzyme Q10, 47. Vitamin E, 48. Vitamin C, 49. Vitamin B12, 51. Fish Oil, 54. Iron, 55. Zinc, 56. Folic Acid, 58. L-Arginine, 60. Probiotics
**Antidepressants (33 questions)**	2. Onion, 6. Red Pepper (Capsicum), 7. Chia seed, 9. Rosemary, 11. Thyme, 16. Aloe Vera, 21. Chamomile, 22. Echinacea, 24. Clove, 29. Blueberry, 30. Horse Chestnut, 31. Licorice, 34. Anise Seed, 37. Fenugreek, 38. Black Cohosh, 39. Burdock, 40. Cat's Claw, 44. Bitter Melon, 45. Devil's Claw, 46. Coenzyme Q10, 47. Vitamin E, 48. Vitamin C, 49. Vitamin B12, 50. Vitamin D, 51. Fish Oil, 52. Calcium, 53. Magnesium, 54. Iron, 55. Zinc, 57. Alpha-Lipoic Acid, 58. L-Arginine, 59. Sports Supplement, 60. Probiotics

A total of 393 questions were evaluated, including 131 questions to assess the level of knowledge about complications and side effects in the surgical period due to the use of nutrients and food supplements, 61 questions to assess the level of knowledge about the duration of cessation of nutrients and food supplements before the surgical period, 201 questions to assess the level of knowledge about nutrients and food supplements and drug interactions. The success score was calculated over 0-100 points based on the number of correct answers of doctors and nurses. Points were calculated for each correct answer. The score for one correct answer (100 points/393 questions≌0.25) was calculated as approximately 0.25 points. Accordingly, the success score for complications and side effects of nutrients and food supplements in the surgical period was 33 points (131*0.25≌33.33), the success score for discontinuation periods before the surgical period was 16 points (61*0.25≌15.52), and the success score for nutrients and food supplements and drug interactions was 51 points (201*0.25≌51.15).

The questions in the questionnaire form were analysed by 5 faculty members who are experts in their fields are to evaluate them in terms of formality, scientific content and comprehensibility criteria. The questionnaire form was finalised in accordance with the form containing the expert opinions.

### Use of data collection tools

Written permission was obtained from the ethics committee of Yıldırım Beyazıt University (06.01.22/36) and the institutions where the research was conducted. Written informed consent was obtained from the doctors and nurses who participated in the research. The study was conducted between 15 February and 31 May 2022, after obtaining ethics committee approval and institutional permission. Nursing directors/health services directors and chief doctors of hospitals with institutional approval were interviewed. The nurse in charge of the surgical clinics and the doctors' clinic chiefs were then interviewed and informed about the study. Information on the working hours, shift patterns and working practices of the doctors and nurses was obtained. To reach more participants, clinic visits were made between 08:00-16:00 and 16:00-20:00. Doctors and nurses were informed about the ethics committee and administrative permissions obtained, the content of the study and the method of implementation. Questions raised by the doctors and nurses were answered. Written informed consent was obtained from doctors and nurses who agreed to participate in the study. The doctors and nurses were interviewed alone in a quiet environment in the doctors' and nurses' room in the clinic. The interview was concluded once the questions had been answered. It took approximately 15-20 minutes for doctors and nurses to complete the questionnaire. The study was completed with 143 doctors and 267 nurses who agreed to participate in the study, a total of 410 participants.

### Analysis of the data

The data obtained by the questionnaire collection method were transferred to the computer environment. The mean, standard deviation, minimum, maximum, median, frequency and percentage were used in the descriptive statistics of the characteristics of the doctors and nurses in the study. Before comparing the knowledge levels of doctors and nurses in groups, the normal distribution status, which is the assumption of parametric analyses, was tested using the Kolmogrow-Smirnow test [85, 86].

Since the groups in the study did not meet the assumption of normal distribution, the Mann-Whitney U test in independent groups was used for pairwise group comparisons, the Kruskal-Wallis H test for three or more group comparisons, and the Mann-Whitney U analysis with Bonferroni correction for post hoc analysis [87–90]. SPSS 26 software was used for statistical analyses [91]. *p*<0.05 was considered statistically significant.

## Results

Table 3 shows that the mean age of the doctors and nurses participating in the study was 33.26 (±7.90) years, 43.2% (*n*=177) were between 22-29 years old, 72% (*n*=295) were female, 46.6% (*n*=191) were graduates, 65. 1% (*n*=267) were nurses, 37.8% (*n*=155) had worked for 10 years or more, 39.3% (*n*=161) worked in general surgery and 39.8% (*n*=163) had worked in their department for 1-4 years. It was found that 88.8% (*n*=364) of doctors and nurses had not received any training on nutrients and food supplements, and 50% of those who had received training on nutrients and food supplements had received it during their professional training.

**Table 3:** Descriptive characteristics of physicians and nurses (n:410)

Descriptive Characteristics		n	%
Age (22-63 years) 33.26 (±7.90)^a^	Between 22-29 Years	**177**	**43.2**
	Between 30-39 Years	151	36.8
	40 years and over	82	20.0
	Total	410	100.0
Sex	Female	**295**	**72.0**
	Male	115	28.0
	Total	410	100.0
Education Level	High School	31	7.6
	Associate Degree	23	5.6
	Graduate Degree	**191**	**46.6**
	Postgraduate Degree	165	40.2
	Total	410	100.0
Occupation	Doctor	143	34.9
	Nurse	**267**	**65.1**
	Total	410	100.0
Years of Work Experience	<5 Years	139	33.9
	5-9 Years	116	28.3
	10 years and over	**155**	**37.8**
	Total	410	100.0
Working Clinic	General Surgery	**161**	**39.3**
	Gynaecology and Obstetrics	45	11
	Cardiovascular Surgery	31	7.6
	Orthopaedics	28	6.8
	Urology	28	6.8
	Plastic and Reconstructive Surgery	28	6.8
	Brain and Neurosurgery	26	6.3
	Ophthalmology	25	6.1
	Otorhinolaryngology	21	5.1
	Thoracic Surgery	17	4.1
	Total	410	100.0
Duration of Service in the Department	<1 Year	103	25.1
	1-4 Years	**163**	**39.8**
	5 Years and more	144	35.1
	Total	410	100.0
Status of Receiving Training on Nutrients and Food Supplements	Yes	46	11.2
	No	**364**	**88.8**
	Total	410	100.0
Place of Training^b^	School of Vocational Education	**24**	**50.00**
	Congress, Symposium, Course	12	25.00
	In-Service Training	11	22.92
	Scientific Publications	8	16.67
	Other	1	2.08

^a^Mean ± SD

^b^Multiple selection

**Fig. 1:**
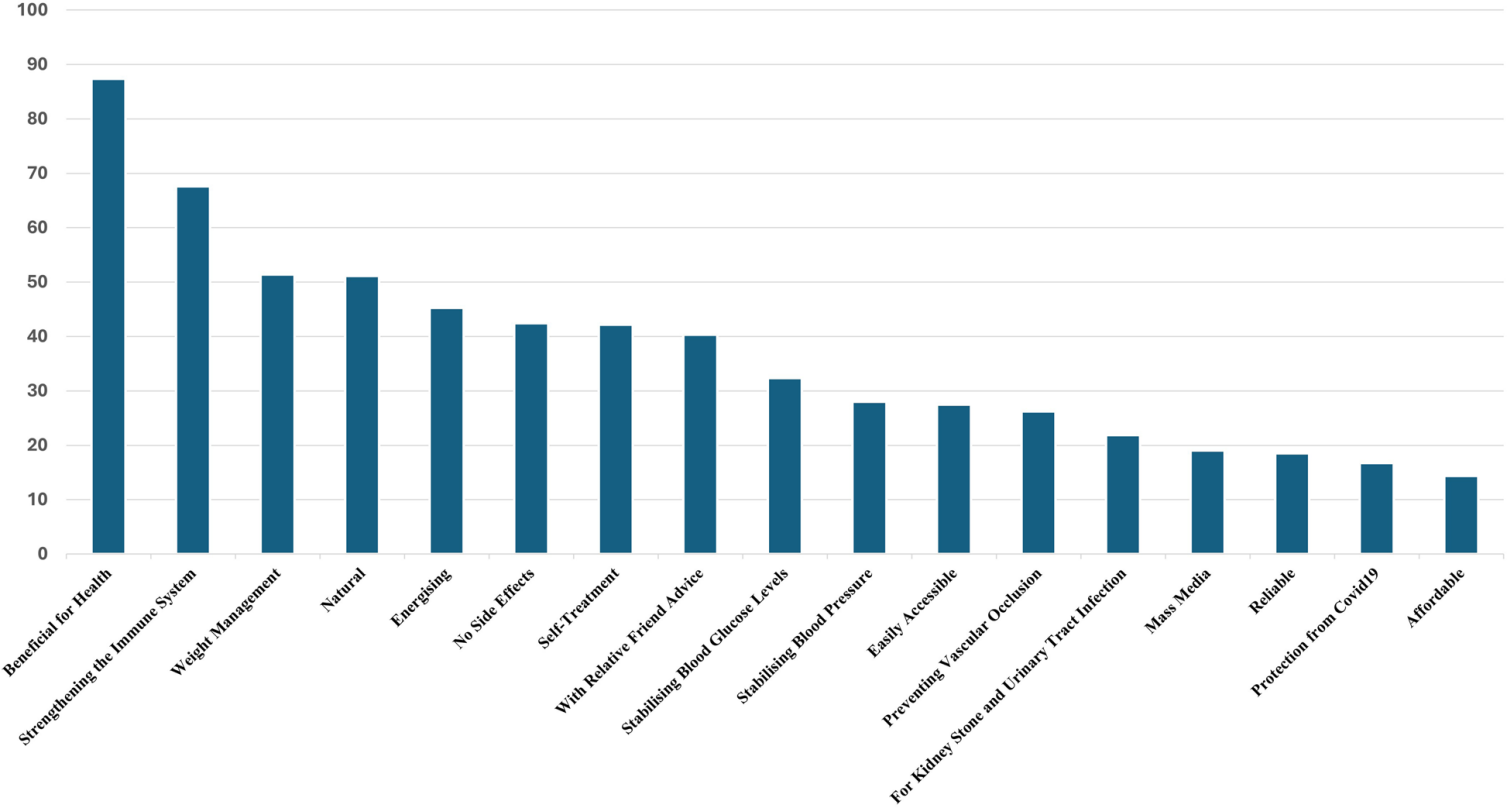
Reasons for patients' preference for nutrients and food supplements according to doctors and nurses

**Fig. 2:**
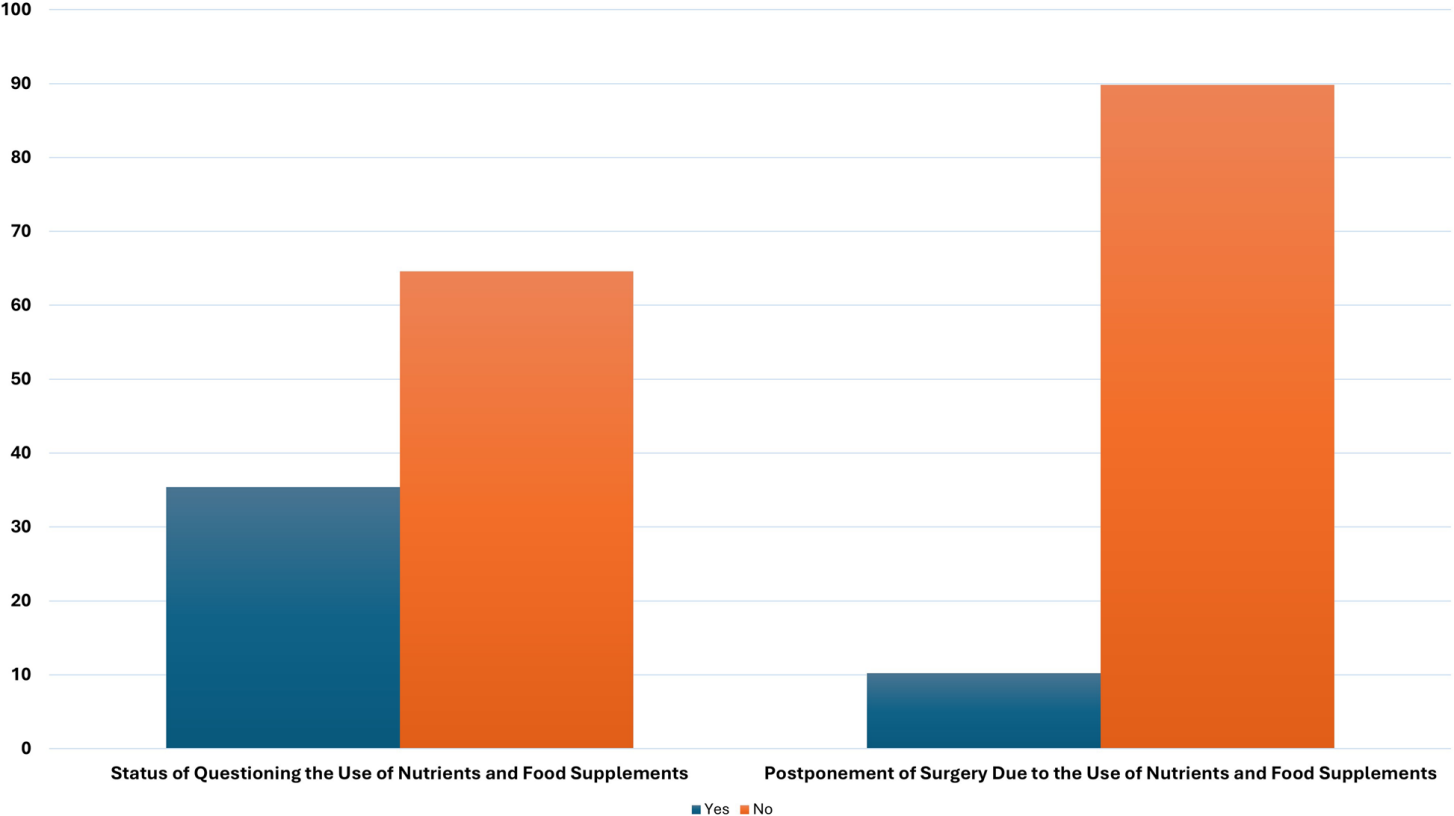
Doctors' and Nurses' Questioning of Patients' Use of Nutrients and Food Supplements and Postponement of Surgery Due to the Use of Nutrients and Food Supplements

Doctors and nurses believed that 87.40% (*n*=340) of patients used nutrients and food supplements because they found them beneficial to their health (Fig. 1). It was found that 64.6% (*n*=265) of doctors and nurses did not question the use of nutrients and food supplements and 89.8% (*n*=368) did not postpone surgery due to the use of nutrients and food supplements (Fig. 2).

The median overall success score of doctors and nurses in the use of nutrients and food supplements was calculated as 3.20. Doctors and nurses received the highest score of 7.06 for complications and side effects of nutrients and food supplements in the surgical period and the lowest score of 0.21 for the duration of discontinuation of nutrients and food supplements prior to the surgical period (Table 4).

**Table 4:** Achievement score distributions of physicians and nurses regarding nutrients and food supplements (n:410)

	Min.	Max.	Med.
Surgical Period Complications and Side Effects (131 questions)	0.00	56.24	7.06
Discontinuation Periods Before Surgical Period (61 questions)	0.00	16.40	0.21
Drug Interactions (201 questions)	0.00	22.00	1.70
Overall Success (393 questions)	0.00	26.50	3.20

A statistically significant difference was found in the success scores of doctors and nurses regarding complications and side effects of nutrients and food supplements in the surgical period in terms of age groups (*p*=0.002), years of employment (*p*=0.025), and receiving training on nutrients and food supplements (*p*<0.05) (Table 5). The knowledge level of the participants in the 22-30 age group was found to be statistically significantly lower than the 31-40 and 41 and over age groups (*p*<0.05). The knowledge level of the participants working for 6-10 years was found to be statistically significantly higher than the participants working for 5 years (*p*<0.05). In addition, the knowledge level of the participants who received training on nutrients and food supplements about the complications and side effects of nutrients and food supplements in the perioperative period was found to be statistically significantly higher than those who did not receive training (*p*<0.05).

**Table 5:** Achievement scores for nutrients and food supplements according to the descriptive characteristics of doctors and nurses (n:410)

		**Complications and Side Effects of Nutrients and Food Supplements Success Score**			**Duration of Nutrients and Food Supplements to be Discontinued Before the Surgical Period Success Score**			**Success Score for Drug Interactions with Nutrients and Food Supplements**			**Total Achievement Score for Nutrients and Food Supplements**		
**Parameters**	**n**	**Median (Min – Max)**	**p**	**Post Hoc.**	**Median (Min – Max)**	**p**	**Post Hoc.**	**Median (Min – Max)**	**p**	**Post Hoc.**	**Median (Min – Max)**	**p**	**Post Hoc.**
**Age (years)**													
**22-30**^**1**^	177	3.04 (0-56.24)	**0.002**^**H**^	1-3** 2-3*	0 (0-13.12)	0.707^**H**^	-	1.0 (0-14)	0.179^**H**^	-	1.5 (0-25.5)	**0.003**^**H**^	2-3**
**30-40**^**2**^	151	4.56 (0-48.64)			0 (0-9.84)			1.0 (0-22)			2.25 (0-26.5)		
**40 and over**^**3**^	82	3.80 (0-3.80)			0 (0-16.40)			1.0 (0-10.50)			1.75 (0-15.50)		
**Sex**													
**Female**	295	3.80 (0-56.24)	0.534^**U**^	-	0 (0-16.40)	0.079^**U**^	-	1.5 (0-1)	**0.001**^**U**^	-	2.025.50)	0.565^**U**^	-
**Male**	115	3.80 (0-48.64)			0 (0-13.12)			0.5 (0-22)			1.50 (0-26.50)		
**Education Level**													
**High School**^**1**^	31	3.04 (0.76-17.48)	0.601^**H**^	-	0 (0-0)	0.670^**H**^	-	1.5 (0-4)	**0.008**^**H**^	1-4*	2.0 (0.25-6.25)	0.992^**H**^	-
**Associate Degree**^**2**^	23	3.04 (0-29.64)			0 (0-16.40)			1.0 (0-10.5)			1.75 (0-15.25)		
**Graduate Degree**^**3**^	191	3.80 (0-56.24)			0 (0-8.20)			1.0 (0-14)			1.75 (0-25.50)		
**Postgraduate Degree**^**4**^	165	3.80 (0-53.96)			0 (0-13.12)			0.5 (0-22)			1.75 (0-26.50)		
**Occupation**													
**Doctor**	143	3.80 (0-53.96)	0.120^**U**^	-	0 (0-13.12)	0.279^**U**^	-	0-22	**0.007**^**U**^	-	1.75 (0-26.5)	0.819^**U**^	-
**Nurse**	267	3.80 (0-56.24)			0 (0-16.40)			0-14			1.75 (0-25.5)		
**Years of Working Experience**													
**<5**^**1**^	139	3.04 (0-53.96)	**0.025**^**H**^	1-2*	0 (0-13.12)	0.055^**H**^	-	0.5 (0-9)	**0.010**^**H**^	1-3*	1.5 (0-20.25)	**0.015**^**H**^	1-2*
**5-10**^**2**^	116	4.56 (0-56.24)			0 (0-9.84)			1.0 (0-15.5)			2.25 (0-25.50)		
**10 years and over**^**3**^	155	3.80 (0-47.12)			0 (0-16.40)			1.0 (0-22)			2.0 (0-26.50)		
**Status of Receiving Training**													
**No**	364	3.80 (0-48.64)	**<0.001**^**U**^	-	0 (0-13.12)	**<0.001**^**U**^	-	1.0 (0-15.5)	**0.032**^**U**^	-	1.1.75 (0-26.5)	**<0.001**^**U**^	-
**Yes**	46	8.36 (0-56.24)			1 (0-16.40)			1.50 (0-22)			3.37 (0-25.5)		

**H:** Kruskal Wallis H Test, **Post-Hoc:** Mann Whitney U Test with Bonferroni Correction at 95% confidence interval, ***p<0.001, **p<0.01, *p<0.05

Success scores related to the duration of discontinuing of nutrients and food supplements in the preoperative period were statistically significantly higher in those who received training on nutrients and food supplements (*p*<0.001) (Table 5).

The success scores of drug interactions of nutrients and food supplements were found to be statistically significantly higher in female participants than in males (*p*=0.001), in nurses than in doctors (*p*=0.007), according to educational status (*p*=0.008), according to years of working experience (*p*=0.010), in those who received training on nutrients and food supplements than in those who did not (*p*<0.05), and in women statistically significantly higher than in men (*p*=0.001) (Table 5). The knowledge level of high school graduates was found to be statistically significantly higher than that of postgraduate participants (*p*<0.05). The knowledge level of the participants working for 11 years or more was found to be statistically significantly higher than the participants working for less than 5 years (*p*<0.05).

The general success scores of doctors and nurses on nutrients and food supplements were found to be statistically significantly higher in older age groups (*p*=0.003), in those who had worked for more years (*p*=0.015) and in those who had received training on nutrients and food supplements (*p*<0.001) (Table 5). The knowledge level of the participants in the 31-40 age group was found to be statistically significantly higher than the participants in the 41 and over age group (*p*<0.01). The knowledge level of the participants who have been working for 6-10 years is statistically significantly higher than the participants who have been working for less than 5 years (*p*<0,05).

## Discussion

Nutrients and food supplements are widely used worldwide [2, 6, 7] for health protection and treatment of chronic diseases [10]. For the same reasons, patients often prefer to use nutrients and food supplements during their surgical period [23, 92]. However, because nutrients and food supplements may contain complex active ingredients with side effects [93, 94], they may cause unexpected complications and side effects [40] or drug interactions in the surgical period [51, 95]. To prevent these complications, side effects and drug interactions, the use of nutrients and food supplements should be stopped two weeks before surgery [39, 44].

Doctors and nurses should ask patients directly about their use of nutrients and food supplements [42] and take a detailed medical history before surgery to prevent potential complications and adverse effects related to nutrients and food supplements during surgery [96]. According to a study conducted by Gamsız et al. (2011), 28.2% of doctors ask patients about their use of nutrients and food supplements during their treatment or before prescribing [97]. Shorofi et al. (2017) found that 15.8% of nurses questioned patients about nutrients and food supplements while taking their medical history [98]. In our study, only 35.4% of doctors and nurses questioned the use of nutrients and food supplements in the preoperative period, and 10.2% reported postponing surgery due to the use of nutrients and food supplements. The average number of operations postponed was 3.72±2.83. The rate of use of nutrients and food supplements during the surgical period in Turkey is 32.5-54.2% [17, 23, 99, 100]. Considering the size of the population in which our study was conducted and the average annual number of operations, the rates of questioning the use of nutrients and food supplements and the number of operations postponed due to the use of nutrients and food supplements were found to be low. This result in our study, which is consistent with the literature, suggests that 11.2% of doctors and nurses have received training in nutrients and food supplements, and the more knowledge doctors and nurses have on this subject, the more they can implement practices aimed at questioning and preventing potential problems.

Doctors and nurses should have a high level of knowledge and awareness of the complications and side effects of nutrients and food supplements in the surgical period, drug interactions [101] and the duration of discontinuation in the preoperative period [35, 39, 44]. However, studies conducted with doctors in the literature emphasise that doctors do not have a sufficient level of knowledge [47, 48]. No studies were found that assessed nurses' knowledge of nutrients and food supplements.

Heller et al (2006) found that 54% of plastic surgeons asked about nutrients and food supplements in the study knew the name and 90% did not know the side effects [102]. Taşpınar et al. (2014) found that 8.6% of doctors correctly answered the duration of stopping nutrients and food supplements in the preoperative period in their study with doctors [103]. Soltanipour et al (2022) found that the mean success score of doctors' (*n*=142) knowledge of nutrients and food supplements was 6.47 ± 6.17 out of 25 points [48]. In this study, the success scores of doctors and nurses related to complications and side effects of nutrients and food supplements were 7.06 out of 33 points, the success scores related to discontinuation periods before the surgical period were 0.21 out of 16 points, the success scores related to drug interactions were 1.70 out of 51 points, and the total success scores were 3.20 out of 100 points. Although our study is consistent with the literature, 88.8% of doctors and nurses did not receive any training on nutrients and food supplements. This situation suggests that nutrients and food supplements are not included in continuing education during professional training or during working period.

Educating doctors and nurses about nutrients and food supplements in both professional and in-service training programmes will increase the knowledge of doctors and nurses [57, 104]. Mikail et al (2003), in their study evaluating doctors' knowledge of nutrients and food supplements, found that the mean pre-test pass rate for doctors was 34% and the mean post-test pass rate after training was 61% [47]. In our study, the overall success score of doctors and nurses who received training on nutrients and food supplements was found to be higher than that of doctors and nurses who did not receive training. In addition, the success scores of doctors and nurses who received training on complications and side effects, discontinuation periods and drug interactions of nutrients and food supplements were also found to be higher. Our results show that education about nutrients and food supplements is effective in improving the level of knowledge.

In addition to education, age and years of experience also influence the level of knowledge of nutrients and food supplements among doctors and nurses [46]. Hasen et al found that doctors, nurses, and pharmacists aged 36-40 years were four times more knowledgeable about nutrients and food supplements than those aged 25-30 years [46]. Nurses' clinical experience and previous patient experience increase the level of knowledge in nursing practice [105].

In this study, it was found that the overall success scores of doctors and nurses in relation to nutrients and food supplements and the success scores in relation to complications and side effects increased statistically significantly with increasing age. In addition, total success scores, success scores related to complications and side effects, and success scores related to drug interactions of nutrients and food supplements increased statistically significantly with increasing years of employment. The results of our study suggest that the experience of doctors and nurses with increasing age and years of employment may contribute positively to the level of knowledge about nutrients and food supplements.

The success score for drug interactions of nurses with a high school degree was found to be statistically significantly higher than that of doctors and nurses with a university degree. This situation suggests that this is due to the statistically significant experience gained from the statistically significantly greater number of years of work of the high school graduate nurses.

Nurses were found to have higher success rates in drug interactions than doctors. This is due to the fact that nurses administer medications, observe side effects, and have experience in this area [106].

In our study, female doctors and nurses were found to have a statistically significant higher success rate with regard to drug interactions with nutrients and food supplements than male doctors and nurses. Koyu et al (2020) evaluated the use of nutrients and food supplements by healthcare professionals throughout the hospital and found that female healthcare professionals used nutrients and food supplements at a higher rate than male healthcare professionals [107]. Taşpınar et al. found that 33.9% of female doctors and 27.5% of male doctors used nutrients and food supplements [103]. It has been reported in the literature that female doctors and nurses prefer nutrients and food supplements more often than male doctors and nurses [103, 107]. The fact that female doctors and nurses had higher achievement scores related to drug interactions of nutrients and food supplements may be related to the higher frequency of use in female doctors and nurses.

In our study, knowledge scores were evaluated in three groups (complications and side effects, drug interactions, discontinuation periods) and as a total score. It was found that the only common and most important factor affecting all three groups and the overall knowledge score was the status of training received on the subject. On the other hand, no positive effect of the level of education (high school, associate degree, undergraduate, postgraduate) on the knowledge level success score was found in any of the knowledge level success score groups. We believe that this situation is due to the lack of training on nutrients and food supplements in current medical and nursing education curricula.

## Conclusion

Although nutrients and food supplements are widely used, in practice they may cause complications and side effects in the surgical period. Therefore, training doctors and nurses on the complications and side effects of nutrients and food supplements in the surgical period, discontinuation periods and drug interactions are necessary to prevent and resolve problems that may occur due to nutrients and food supplements used by the patient. In order to prevent potential complications and side effects in the surgical period due to misuse of nutrients and food supplements, it is essential that doctors and nurses are educated and have a high awareness of the use of nutrients and food supplements. In this study, it was found that the level of knowledge of nutrients and food supplements among doctors and nurses working in surgical clinics was less than half or even close to zero. This study revealed significant gaps in physicians' and nurses' knowledge of nutrients and food supplements and highlighted the need for comprehensive education to prevent potential risks associated with the use of nutrients and food supplements during the surgical period.

### Supplementary Information

**Supplementary Material 1.** 

## Data Availability

The datasets generated during and/or analysed during the current study are available in the Zonedo repository, 10.5281/zenodo.10738144 [108]. Büyükkasap AE, Yazıcı G. Knowledge levels of doctors and nurses working in surgical clinics about nutrients and food supplements, a multicentre descriptive study, dataset. Zenodo. 2024. 10.5281/zenodo.10738144 The research used a questionnaire-interview method to collect data. This questionnaire-interview was designed for this study by the researchers utilizing the literature (Supplementary Material 1).
