# Supplementary material for: Knowledge levels of doctors and nurses working in surgical clinics about nutrients and food supplements, a multicentre descriptive study
Source: BMC Nurs. 2024 Apr 25;23:277. doi: 10.1186/s12912-024-01968-z (PMC11044485; doi:10.1186/s12912-024-01968-z)
Supplement: Supplementary file 1 — Supplementary Material 1. [file 12912_2024_1968_MOESM1_ESM.docx]

**Knowledge levels of doctors and nurses working in surgical clinics about nutrients and food supplements, a multicentre descriptive study**

*Section 1. Socio-Demographic Characteristics*

1. Age :……………………………………………………………………………………..
2. Gender: ( ) Female ( ) Male
3. Level of Education: ( ) High School ( ) Associate Degree ( ) Bachelor's Degree ( ) Graduate Degree
4. Marital Status: ( ) Married ( ) Single
5. Occupation: ( ) Physician ( ) Nurse
6. Total Years of Professional Work:....................................................................
7. Department you work in:.................................................................................
8. Years working in your current department:..........................................................
9. Have you received any training on Nutrients and food supplements?

( ) Yes ( ) No

1. If your answer is "Yes", where did you receive the training?

( ) During the vocational training period

( ) Congress, Symposium etc.

( ) From in-service training

( ) From scientific publications

( ) Other……………………………………

1. Why do you think patients prefer Nutrients and food supplements? (You can select more than one option.)

( ) Because they find it beneficial to health

( ) Because they think it balances blood pressure

( ) Because they think it prevents vascular occlusion

( ) To balance blood glucose level

( ) For the treatment of kidney stones and urinary tract infection

( ) For weight control

( ) Because they think it gives energy

( ) Because they think it strengthens the immune system

( ) Because they find it reliable

( ) Because they find it natural

( ) Because they think it is easily accessible

( ) Because they think it has no side effects

( ) Because they found it cheap

( ) Because they have the chance to treat themselves

( ) Other:…………………………………………………………………

1. Do you question patients' use of nutrients and food supplements?

( ) Yes ( ) No

1. Have you ever postponed surgery due to the use of nutrients and food supplements?

( ) Yes ( ) No

1. If your answer is yes, how many patients have had it?........................................................ ........................

*Section 2. Determination of Knowledge Levels Regarding Complications and Side Effects Seen in the Perioperative Period Due to the Use of nutrients and food supplements*

1. In the postoperative period, many complications and side effects may occur due to the use of nutrients and food supplements. The most common complications and side effects are listed below. According to this, which nutrient and food supplement causes which complication and side effect in your opinion? Please mark.

| Nutrients and food supplements | Bleeding | | | | Fluid-Electrolyte Imbalance | | | Hepatotoxic Effect | | | Blood Glucose Level | | | |
| --- | --- | --- | --- | --- | --- | --- | --- | --- | --- | --- | --- | --- | --- | --- |
|  | Increases | Reduces | Does not affect | I dont know | Does | Does not affect | I dont know | Does | Does not affect | I dont know | Increases | Reduces | Does not affect | I dont know |
| 1. Garlic |  |  |  |  |  |  |  |  |  |  |  |  |  |  |
| 1. Onion |  |  |  |  |  |  |  |  |  |  |  |  |  |  |
| 1. Lemon |  |  |  |  |  |  |  |  |  |  |  |  |  |  |
| 1. Nettle |  |  |  |  |  |  |  |  |  |  |  |  |  |  |
| 1. Parsley |  |  |  |  |  |  |  |  |  |  |  |  |  |  |
| 1. Red Pepper (Capsicum) |  |  |  |  |  |  |  |  |  |  |  |  |  |  |
| 1. Chia Seed |  |  |  |  |  |  |  |  |  |  |  |  |  |  |
| 1. Celery |  |  |  |  |  |  |  |  |  |  |  |  |  |  |
| 1. Rosemary |  |  |  |  |  |  |  |  |  |  |  |  |  |  |
| 1. Sage |  |  |  |  |  |  |  |  |  |  |  |  |  |  |
| 1. Thyme |  |  |  |  |  |  |  |  |  |  |  |  |  |  |
| 1. St. John's Wort |  |  |  |  |  |  |  |  |  |  |  |  |  |  |
| 1. Turmeric |  |  |  |  |  |  |  |  |  |  |  |  |  |  |
| 1. Ginger |  |  |  |  |  |  |  |  |  |  |  |  |  |  |
| 1. Hawthorn |  |  |  |  |  |  |  |  |  |  |  |  |  |  |
| 1. Aloe vera |  |  |  |  |  |  |  |  |  |  |  |  |  |  |
| 1. Black tea |  |  |  |  |  |  |  |  |  |  |  |  |  |  |
| 1. Green tea |  |  |  |  |  |  |  |  |  |  |  |  |  |  |
| 1. Linden |  |  |  |  |  |  |  |  |  |  |  |  |  |  |
| 1. Lavender |  |  |  |  |  |  |  |  |  |  |  |  |  |  |
| 1. Chamomile |  |  |  |  |  |  |  |  |  |  |  |  |  |  |
| 1. Echinacea |  |  |  |  |  |  |  |  |  |  |  |  |  |  |
| 1. Calendula |  |  |  |  |  |  |  |  |  |  |  |  |  |  |
| 1. Cloves |  |  |  |  |  |  |  |  |  |  |  |  |  |  |
| 1. Dandelion |  |  |  |  |  |  |  |  |  |  |  |  |  |  |
| 1. Ginseng |  |  |  |  |  |  |  |  |  |  |  |  |  |  |
| 1. Ginkgo Biloba |  |  |  |  |  |  |  |  |  |  |  |  |  |  |
| 1. Cherry |  |  |  |  |  |  |  |  |  |  |  |  |  |  |
| 1. Blueberries |  |  |  |  |  |  |  |  |  |  |  |  |  |  |
| 1. Horse Chestnut |  |  |  |  |  |  |  |  |  |  |  |  |  |  |
| 1. Liquorice |  |  |  |  |  |  |  |  |  |  |  |  |  |  |
| 1. Valerian |  |  |  |  |  |  |  |  |  |  |  |  |  |  |
| 1. Red Clover |  |  |  |  |  |  |  |  |  |  |  |  |  |  |
| 1. Anise Seed |  |  |  |  |  |  |  |  |  |  |  |  |  |  |
| 1. Ephedra |  |  |  |  |  |  |  |  |  |  |  |  |  |  |
| 1. Kava |  |  |  |  |  |  |  |  |  |  |  |  |  |  |
| 1. Fenugreek |  |  |  |  |  |  |  |  |  |  |  |  |  |  |
| 1. Black Cohosh |  |  |  |  |  |  |  |  |  |  |  |  |  |  |
| 1. Burdock |  |  |  |  |  |  |  |  |  |  |  |  |  |  |
| 1. Cat's Claw |  |  |  |  |  |  |  |  |  |  |  |  |  |  |
| 1. Mistletoe |  |  |  |  |  |  |  |  |  |  |  |  |  |  |
| 1. Hops |  |  |  |  |  |  |  |  |  |  |  |  |  |  |
| 1. Passionflower |  |  |  |  |  |  |  |  |  |  |  |  |  |  |
| 1. Bitter Melon |  |  |  |  |  |  |  |  |  |  |  |  |  |  |
| 1. Devil's Claw |  |  |  |  |  |  |  |  |  |  |  |  |  |  |
| 1. Coenzyme Q10 |  |  |  |  |  |  |  |  |  |  |  |  |  |  |
| 1. Vitamin E |  |  |  |  |  |  |  |  |  |  |  |  |  |  |
| 1. Vitamin C |  |  |  |  |  |  |  |  |  |  |  |  |  |  |
| 1. Vitamin B12 |  |  |  |  |  |  |  |  |  |  |  |  |  |  |
| 1. Vitamin D |  |  |  |  |  |  |  |  |  |  |  |  |  |  |
| 1. Fish oil |  |  |  |  |  |  |  |  |  |  |  |  |  |  |
| 1. Calcium |  |  |  |  |  |  |  |  |  |  |  |  |  |  |
| 1. Magnesium |  |  |  |  |  |  |  |  |  |  |  |  |  |  |
| 1. Iron |  |  |  |  |  |  |  |  |  |  |  |  |  |  |
| 1. Zinc |  |  |  |  |  |  |  |  |  |  |  |  |  |  |
| 1. Folic acid |  |  |  |  |  |  |  |  |  |  |  |  |  |  |
| 1. Alpha-Lipoic Acid |  |  |  |  |  |  |  |  |  |  |  |  |  |  |
| 1. L-Arginine |  |  |  |  |  |  |  |  |  |  |  |  |  |  |
| 1. Sports Supplement |  |  |  |  |  |  |  |  |  |  |  |  |  |  |
| 1. Probiotics |  |  |  |  |  |  |  |  |  |  |  |  |  |  |
| 1. Weight-Loss Products |  |  |  |  |  |  |  |  |  |  |  |  |  |  |

*Section 3: Determination of Knowledge Levels Regarding Preoperative Discontinuation of Nutrients and Food supplements*

16. In your opinion, what is the duration of discontinuation of the following nutrients and food supplements in the preoperative period? If you know the answer and think that it should be stopped "... If you think it should be cut a day before,” you can write one of the numbers between “6 and 1” in that section, “…. If you think "it should be cut off hours ago", you can write one of the numbers between "23 and 1" in that section. If you do not know the answer, you can mark the "I do not know the answer" section.

| Nutritional and Food Supplement Product | Preoperative Discontinuation of Nutrients and Food supplements | | | |
| --- | --- | --- | --- | --- |
|  | It should be discontinued … weeks before surgery. | It should be discontinued … days before surgery.  (period less than seven days will be written) | It should be discontinued … hours before surgery.  (period less than twenty-four hours will be written) | I dont know |
| 1. Garlic |  |  |  |  |
| 1. Onion |  |  |  |  |
| 1. Lemon |  |  |  |  |
| 1. Nettle |  |  |  |  |
| 1. Parsley |  |  |  |  |
| 1. Red Pepper (Capsicum) |  |  |  |  |
| 1. Chia Seed |  |  |  |  |
| 1. Celery |  |  |  |  |
| 1. Rosemary |  |  |  |  |
| 1. Sage |  |  |  |  |
| 1. Thyme |  |  |  |  |
| 1. St. John's Wort |  |  |  |  |
| 1. Turmeric |  |  |  |  |
| 1. Ginger |  |  |  |  |
| 1. Hawthorn |  |  |  |  |
| 1. Aloe vera |  |  |  |  |
| 1. Black tea |  |  |  |  |
| 1. Green tea |  |  |  |  |
| 1. Linden |  |  |  |  |
| 1. Lavender |  |  |  |  |
| 1. Chamomile |  |  |  |  |
| 1. Echinacea |  |  |  |  |
| 1. Calendula |  |  |  |  |
| 1. Cloves |  |  |  |  |
| 1. Dandelion |  |  |  |  |
| 1. Ginseng |  |  |  |  |
| 1. Ginkgo Biloba |  |  |  |  |
| 1. Cherry |  |  |  |  |
| 1. Blueberries |  |  |  |  |
| 1. Horse Chestnut |  |  |  |  |
| 1. Liquorice |  |  |  |  |
| 1. Valerian |  |  |  |  |
| 1. Red Clover |  |  |  |  |
| 1. Anise Seed |  |  |  |  |
| 1. Ephedra |  |  |  |  |
| 1. Kava |  |  |  |  |
| 1. Fenugreek |  |  |  |  |
| 1. Black Cohosh |  |  |  |  |
| 1. Burdock |  |  |  |  |
| 1. Cat's Claw |  |  |  |  |
| 1. Mistletoe |  |  |  |  |
| 1. Hops |  |  |  |  |
| 1. Passionflower |  |  |  |  |
| 1. Bitter Melon |  |  |  |  |
| 1. Devil's Claw |  |  |  |  |
| 1. Coenzyme Q10 |  |  |  |  |
| 1. Vitamin E |  |  |  |  |
| 1. Vitamin C |  |  |  |  |
| 1. Vitamin B12 |  |  |  |  |
| 1. Vitamin D |  |  |  |  |
| 1. Fish oil |  |  |  |  |
| 1. Calcium |  |  |  |  |
| 1. Magnesium |  |  |  |  |
| 1. Iron |  |  |  |  |
| 1. Zinc |  |  |  |  |
| 1. Folic acid |  |  |  |  |
| 1. Alpha-Lipoic Acid |  |  |  |  |
| 1. L-Arginine |  |  |  |  |
| 1. Sports Supplement |  |  |  |  |
| 1. Probiotics |  |  |  |  |
| 1. Weight-Loss Products |  |  |  |  |

*Section 4: Evaluation of Knowledge Levels Regarding Drug Interactions of Nutrients and food supplements*

17. The nutrients and food supplements listed below interact with many drug groups. The drug groups given in the table are the ones that interact most with nutrients and food supplements when looking at the literature. How do you think the nutrients and food supplements given in the table affect these drug groups? Please mark it.

| Effectiveness of … | | Antihypertensives | | | | Anticoagulants | | | | | Anesthetics | | | | | Analgesics | | | | | Corticosteroids | | | | | Antidiabetics | | | | | Antidepressants | | | | |
| --- | --- | --- | --- | --- | --- | --- | --- | --- | --- | --- | --- | --- | --- | --- | --- | --- | --- | --- | --- | --- | --- | --- | --- | --- | --- | --- | --- | --- | --- | --- | --- | --- | --- | --- | --- |
| Nutritional and Food Supplement Product | | Increases | Reduces | Does not affect | I dont know | Increases | Reduces | Does not affect | I dont know | Increases | | Reduces | Does not affect | I dont know | Increases | | Reduces | Does not affect | I dont know | Increases | | Reduces | Does not affect | I dont know | Increases | | Reduces | Does not affect | I dont know | Increases | | Reduces | Does not affect | I dont know |  |
| 1. 11 | Garlic |  |  |  |  |  |  |  |  |  | |  |  |  |  | |  |  |  |  | |  |  |  |  | |  |  |  |  | |  |  |  |  |
|  | Onion |  |  |  |  |  |  |  |  |  | |  |  |  |  | |  |  |  |  | |  |  |  |  | |  |  |  |  | |  |  |  |  |
|  | Lemon |  |  |  |  |  |  |  |  |  | |  |  |  |  | |  |  |  |  | |  |  |  |  | |  |  |  |  | |  |  |  |  |
|  | Nettle |  |  |  |  |  |  |  |  |  | |  |  |  |  | |  |  |  |  | |  |  |  |  | |  |  |  |  | |  |  |  |  |
|  | Parsley |  |  |  |  |  |  |  |  |  | |  |  |  |  | |  |  |  |  | |  |  |  |  | |  |  |  |  | |  |  |  |  |
|  | Red Pepper (Capsicum) |  |  |  |  |  |  |  |  |  | |  |  |  |  | |  |  |  |  | |  |  |  |  | |  |  |  |  | |  |  |  |  |
|  | Chia Seed |  |  |  |  |  |  |  |  |  | |  |  |  |  | |  |  |  |  | |  |  |  |  | |  |  |  |  | |  |  |  |  |
|  | Celery |  |  |  |  |  |  |  |  |  | |  |  |  |  | |  |  |  |  | |  |  |  |  | |  |  |  |  | |  |  |  |  |
|  | Rosemary |  |  |  |  |  |  |  |  |  | |  |  |  |  | |  |  |  |  | |  |  |  |  | |  |  |  |  | |  |  |  |  |
|  | Sage |  |  |  |  |  |  |  |  |  | |  |  |  |  | |  |  |  |  | |  |  |  |  | |  |  |  |  | |  |  |  |  |
|  | Thyme |  |  |  |  |  |  |  |  |  | |  |  |  |  | |  |  |  |  | |  |  |  |  | |  |  |  |  | |  |  |  |  |
|  | St. John's Wort |  |  |  |  |  |  |  |  |  | |  |  |  |  | |  |  |  |  | |  |  |  |  | |  |  |  |  | |  |  |  |  |
|  | Turmeric |  |  |  |  |  |  |  |  |  | |  |  |  |  | |  |  |  |  | |  |  |  |  | |  |  |  |  | |  |  |  |  |
|  | Ginger |  |  |  |  |  |  |  |  |  | |  |  |  |  | |  |  |  |  | |  |  |  |  | |  |  |  |  | |  |  |  |  |
|  | Hawthorn |  |  |  |  |  |  |  |  |  | |  |  |  |  | |  |  |  |  | |  |  |  |  | |  |  |  |  | |  |  |  |  |
|  | Aloe vera |  |  |  |  |  |  |  |  |  | |  |  |  |  | |  |  |  |  | |  |  |  |  | |  |  |  |  | |  |  |  |  |
|  | Black tea |  |  |  |  |  |  |  |  |  | |  |  |  |  | |  |  |  |  | |  |  |  |  | |  |  |  |  | |  |  |  |  |
|  | Green tea |  |  |  |  |  |  |  |  |  | |  |  |  |  | |  |  |  |  | |  |  |  |  | |  |  |  |  | |  |  |  |  |
|  | Linden |  |  |  |  |  |  |  |  |  | |  |  |  |  | |  |  |  |  | |  |  |  |  | |  |  |  |  | |  |  |  |  |
|  | Lavender |  |  |  |  |  |  |  |  |  | |  |  |  |  | |  |  |  |  | |  |  |  |  | |  |  |  |  | |  |  |  |  |
|  | Chamomile |  |  |  |  |  |  |  |  |  | |  |  |  |  | |  |  |  |  | |  |  |  |  | |  |  |  |  | |  |  |  |  |
|  | Echinacea |  |  |  |  |  |  |  |  |  | |  |  |  |  | |  |  |  |  | |  |  |  |  | |  |  |  |  | |  |  |  |  |
|  | Calendula |  |  |  |  |  |  |  |  |  | |  |  |  |  | |  |  |  |  | |  |  |  |  | |  |  |  |  | |  |  |  |  |
|  | Cloves |  |  |  |  |  |  |  |  |  | |  |  |  |  | |  |  |  |  | |  |  |  |  | |  |  |  |  | |  |  |  |  |
|  | Dandelion |  |  |  |  |  |  |  |  |  | |  |  |  |  | |  |  |  |  | |  |  |  |  | |  |  |  |  | |  |  |  |  |
|  | Ginseng |  |  |  |  |  |  |  |  |  | |  |  |  |  | |  |  |  |  | |  |  |  |  | |  |  |  |  | |  |  |  |  |
|  | Ginkgo Biloba |  |  |  |  |  |  |  |  |  | |  |  |  |  | |  |  |  |  | |  |  |  |  | |  |  |  |  | |  |  |  |  |
|  | Cherry |  |  |  |  |  |  |  |  |  | |  |  |  |  | |  |  |  |  | |  |  |  |  | |  |  |  |  | |  |  |  |  |
|  | Blueberries |  |  |  |  |  |  |  |  |  | |  |  |  |  | |  |  |  |  | |  |  |  |  | |  |  |  |  | |  |  |  |  |
|  | Horse Chestnut |  |  |  |  |  |  |  |  |  | |  |  |  |  | |  |  |  |  | |  |  |  |  | |  |  |  |  | |  |  |  |  |
|  | Liquorice |  |  |  |  |  |  |  |  |  | |  |  |  |  | |  |  |  |  | |  |  |  |  | |  |  |  |  | |  |  |  |  |
|  | Valerian |  |  |  |  |  |  |  |  |  | |  |  |  |  | |  |  |  |  | |  |  |  |  | |  |  |  |  | |  |  |  |  |
|  | Red Clover |  |  |  |  |  |  |  |  |  | |  |  |  |  | |  |  |  |  | |  |  |  |  | |  |  |  |  | |  |  |  |  |
|  | Anise Seed |  |  |  |  |  |  |  |  |  | |  |  |  |  | |  |  |  |  | |  |  |  |  | |  |  |  |  | |  |  |  |  |
|  | Ephedra |  |  |  |  |  |  |  |  |  | |  |  |  |  | |  |  |  |  | |  |  |  |  | |  |  |  |  | |  |  |  |  |
|  | Kava |  |  |  |  |  |  |  |  |  | |  |  |  |  | |  |  |  |  | |  |  |  |  | |  |  |  |  | |  |  |  |  |
|  | Fenugreek |  |  |  |  |  |  |  |  |  | |  |  |  |  | |  |  |  |  | |  |  |  |  | |  |  |  |  | |  |  |  |  |
|  | Black Cohosh |  |  |  |  |  |  |  |  |  | |  |  |  |  | |  |  |  |  | |  |  |  |  | |  |  |  |  | |  |  |  |  |
|  | Burdock |  |  |  |  |  |  |  |  |  | |  |  |  |  | |  |  |  |  | |  |  |  |  | |  |  |  |  | |  |  |  |  |
|  | Cat's Claw |  |  |  |  |  |  |  |  |  | |  |  |  |  | |  |  |  |  | |  |  |  |  | |  |  |  |  | |  |  |  |  |
|  | Mistletoe |  |  |  |  |  |  |  |  |  | |  |  |  |  | |  |  |  |  | |  |  |  |  | |  |  |  |  | |  |  |  |  |
|  | Hops |  |  |  |  |  |  |  |  |  | |  |  |  |  | |  |  |  |  | |  |  |  |  | |  |  |  |  | |  |  |  |  |
|  | Passionflower |  |  |  |  |  |  |  |  |  | |  |  |  |  | |  |  |  |  | |  |  |  |  | |  |  |  |  | |  |  |  |  |
|  | Bitter Melon |  |  |  |  |  |  |  |  |  | |  |  |  |  | |  |  |  |  | |  |  |  |  | |  |  |  |  | |  |  |  |  |
|  | Devil's Claw |  |  |  |  |  |  |  |  |  | |  |  |  |  | |  |  |  |  | |  |  |  |  | |  |  |  |  | |  |  |  |  |
|  | Coenzyme Q10 |  |  |  |  |  |  |  |  |  | |  |  |  |  | |  |  |  |  | |  |  |  |  | |  |  |  |  | |  |  |  |  |
|  | Vitamin E |  |  |  |  |  |  |  |  |  | |  |  |  |  | |  |  |  |  | |  |  |  |  | |  |  |  |  | |  |  |  |  |
|  | Vitamin C |  |  |  |  |  |  |  |  |  | |  |  |  |  | |  |  |  |  | |  |  |  |  | |  |  |  |  | |  |  |  |  |
|  | Vitamin B12 |  |  |  |  |  |  |  |  |  | |  |  |  |  | |  |  |  |  | |  |  |  |  | |  |  |  |  | |  |  |  |  |
|  | Vitamin D |  |  |  |  |  |  |  |  |  | |  |  |  |  | |  |  |  |  | |  |  |  |  | |  |  |  |  | |  |  |  |  |
|  | Fish oil |  |  |  |  |  |  |  |  |  | |  |  |  |  | |  |  |  |  | |  |  |  |  | |  |  |  |  | |  |  |  |  |
|  | Calcium |  |  |  |  |  |  |  |  |  | |  |  |  |  | |  |  |  |  | |  |  |  |  | |  |  |  |  | |  |  |  |  |
|  | Magnesium |  |  |  |  |  |  |  |  |  | |  |  |  |  | |  |  |  |  | |  |  |  |  | |  |  |  |  | |  |  |  |  |
|  | Iron |  |  |  |  |  |  |  |  |  | |  |  |  |  | |  |  |  |  | |  |  |  |  | |  |  |  |  | |  |  |  |  |
|  | Zinc |  |  |  |  |  |  |  |  |  | |  |  |  |  | |  |  |  |  | |  |  |  |  | |  |  |  |  | |  |  |  |  |
|  | Folic acid |  |  |  |  |  |  |  |  |  | |  |  |  |  | |  |  |  |  | |  |  |  |  | |  |  |  |  | |  |  |  |  |
|  | Alpha-Lipoic Acid |  |  |  |  |  |  |  |  |  | |  |  |  |  | |  |  |  |  | |  |  |  |  | |  |  |  |  | |  |  |  |  |
|  | L-Arginine |  |  |  |  |  |  |  |  |  | |  |  |  |  | |  |  |  |  | |  |  |  |  | |  |  |  |  | |  |  |  |  |
|  | Sports Supplement |  |  |  |  |  |  |  |  |  | |  |  |  |  | |  |  |  |  | |  |  |  |  | |  |  |  |  | |  |  |  |  |
|  | Probiotics |  |  |  |  |  |  |  |  |  | |  |  |  |  | |  |  |  |  | |  |  |  |  | |  |  |  |  | |  |  |  |  |
|  | Weight-Loss Products |  |  |  |  |  |  |  |  |  | |  |  |  |  | |  |  |  |  | |  |  |  |  | |  |  |  |  | |  |  |  |  |
